# Lightweight Sheep Face Recognition Model Combining Grouped Convolution and Parameter Fusion

**DOI:** 10.3390/s25154610

**Published:** 2025-07-25

**Authors:** Gaochao Liu, Lijun Kang, Yongqiang Dai

**Affiliations:** College of Information Science and Technology, Gansu Agricultural University, Lanzhou 730070, China; liugc@st.gsau.edu.cn (G.L.); dyq@gsau.edu.cn (Y.D.)

**Keywords:** sheep face recognition, YOLOv8n, model lightweight, attention mechanism

## Abstract

**Highlights:**

**What are the main findings?**

- Proposed PFL-YOLO, a lightweight deep learning model specifically designed for sheep face recognition, integrating efficient feature extraction strategies.
- Achieved high-precision sheep face recognition with low computational complexity, balancing accuracy and efficiency effectively.

**What is the implication of the main finding?**

- Outperforms mainstream recognition models in extracting frontal face features of sheep, demonstrating superior performance in livestock monitoring.
- Enables feasible deployment on mobile devices, embedded systems, and surveillance networks, promoting practical applications in intelligent agriculture.

**Abstract:**

Sheep face recognition technology is critical in key areas such as individual sheep identification and behavior monitoring. Existing sheep face recognition models typically require high computational resources. When these models are deployed on mobile or embedded devices, problems such as reduced model recognition accuracy and increased recognition time arise. To address these problems, an improved Parameter Fusion Lightweight You Only Look Once (PFL-YOLO) sheep face recognition model based on YOLOv8n is proposed. In this study, the Efficient Hybrid Conv (EHConv) module is first integrated to enhance the extraction capability of the model for sheep face features. At the same time, the Residual C2f (RC2f) module is introduced to facilitate the effective fusion of multi-scale feature information and improve the information processing capability of the model; furthermore, the Efficient Spatial Pyramid Pooling Fast (ESPPF) module was used to fuse features of different scales. Finally, parameter fusion optimization work was carried out for the detection head, and the construction of the Parameter Fusion Detection (PFDetect) module was achieved, which significantly reduced the number of model parameters and computational complexity. The experimental results show that the PFL-YOLO model exhibits an excellent performance–efficiency balance in sheep face recognition tasks: mAP@50 and mAP@50:95 reach 99.5% and 87.4%, respectively, and the accuracy is close to or equal to the mainstream benchmark model. At the same time, the number of parameters is only 1.01 M, which is reduced by 45.1%, 83.7%, 66.6%, 71.4%, and 61.2% compared to YOLOv5n, YOLOv7-tiny, YOLOv8n, YOLOv9-t, and YOLO11n, respectively. The size of the model was compressed to 2.1 MB, which was reduced by 44.7%, 82.5%, 65%, 72%, and 59.6%, respectively, compared to similar lightweight models. The experimental results confirm that the PFL-YOLO model maintains high accuracy recognition performance while being lightweight and can provide a new solution for sheep face recognition models on resource-constrained devices.

## 1. Introduction

In today’s era of rapid technological development, intelligent agriculture has become an important trend in the field of agriculture. Castañeda-Miranda et al. and Chatterjee et al. utilize IoT technology to achieve smart agriculture [1,2]. Castañeda-Miranda et al. employ IoT technology and artificial neural networks to monitor and collect environmental data in real time, enabling intelligent frost control and irrigation management [1]. Chatterjee et al. have developed an IoT-based livestock health monitoring framework that can identify the health status of dairy cows and analyze changes in their behavior to predict various dairy cow diseases [2]. IoT technology has significantly improved the efficiency of agricultural systems. Animal husbandry, as a pillar industry of agriculture, is being promoted by the Internet of Things (IoT), artificial intelligence, and other technologies for precise and intelligent management. Zhang et al. discussed the application of Wearable Internet of Things (W-IoT) technology in smart farms to realize precision livestock breeding, where accurate identification of individual animals is the key to realizing precision management [3]. Many scholars have used deep learning technology to perform facial recognition on pigs and cattle to achieve accurate individual identification [4,5]. Deep learning-driven animal facial recognition technology (such as individual identification of livestock, such as pigs and cattle) is driving the livestock industry toward a paradigm shift toward individual precision management, intelligent health monitoring, and automated behavioral analysis, providing core technological support for agricultural intelligence. For sheep, as a widely farmed livestock species worldwide, the accurate identification of its individual animals is of great significance to improve farming efficiency and ensure product safety.

Traditional identification of individual sheep relies mainly on physical marking and manual observation: ear tags are easily removed, branding can cause infection, and spray paint fails as the wool grows; the Radio Frequency Identification (RFID) [6] technology allows for electronic identification but suffers from the risk of tag damage and high cost, while empirical body observation methods are inefficient and difficult to scale up. These methods have significant limitations in terms of durability, automation, and affordability. In contrast, biometric technologies are more adaptable: DNA detection is difficult to scale up due to the lack of real-time performance and high cost, iris recognition is limited by equipment investment and animal cooperation, and facial recognition can analyze sheep’s facial features using deep learning technology to identify individuals, which can simultaneously meet the demand for accurate management and tracking of health data, providing an efficient and reliable solution for modern agriculture.

With the rapid development of deep learning technology, sheep face recognition shows important application value in intelligent animal husbandry. The existing research mainly focuses on four directions: Convolutional Neural Networks (CNNs), Vision Transformer (ViT), Metric Learning, and model lightweighting. Although each method has made significant progress in specific scenarios, there is still room for improvement in terms of model efficiency, feature representation capability, and complex scene adaptation. Early research focused on building end-to-end recognition frameworks using CNNs. Almog et al. collected 81 Assaf goat facial images, first localized the goat face with Faster R-CNN, and then classified it with ResNet50V2 model embedded with ArcFace loss function, with an average accuracy of 95% on the two datasets, but the number of parameters was 40.4 M [7]. Billah et al. used a YOLOv4 model to recognize the key parts of a goat’s face; the accuracy of face, eyes, ears, and nose was 93%, 83%, 92%, and 85%; however, global facial features are not used, and there may be missed detections [8]. Zhang et al. used the YOLOv4 model, incorporating the CBAM attention mechanism to recognize the sheep’s face trained by migration learning; the mAP@50 was 91.58% and 90.61% in the two datasets, respectively, demonstrating the importance of the attention mechanism in screening key features. However, the number of parameters and floating-point operations of YOLOv4 are extremely large, which makes it difficult to meet the requirements of mobile deployment [9]. Zhang et al. incorporated the SE attention mechanism into the AlexNet model and used the Mish activation function. The recognition accuracy on the validation set reached 98.37%, and the accuracy on the validation set was about 96.58% after 100 days of tracking and collection. It demonstrates the long-term stability of the model, but its shallow network structure limits the ability to handle complex occlusion scenarios [10]. Guo et al. collected 10 types of sheep facial images as a dataset. They used knowledge distillation to migrate the knowledge from YOLOv5x to YOLOv5s to enhance the feature extraction capability, with a model accuracy of 92.75%, mAP@50:95 of 94.67%, and an inference time of 12.63 ms. However, the number of model parameters is 7.2 M, and the model size is 14.065 MB, which is still far from the lightweight model [11]. Pang et al. proposed the Attention Residual Module (ARM) on a self-constructed dataset containing 4490 images of 38 sheep with VGG16, GoogLeNet, and ResNet50, combined with 10.2%, 6.65%, and 4.38% accuracy improvement, respectively. However, the number of ARM module references is 1.6 M, which may not satisfy the lightweight requirement in combination with other models [12].

Many other researchers have used the algorithm based on the Vision Transformer architecture to achieve good results in sheep face recognition. Li et al. combined MobileNetV2 with ViT. They achieved 97.13% accuracy on a dataset of 7434 images containing 186 sheep, with the number of parameters and floating-point operations reduced by 5 times compared to that of ResNet-50 [13]. Zhang et al. improved the ViT model by introducing the LayerScale module and migration learning method, achieving 97.9% accuracy on a self-built dataset of 16,000 images containing 160 sheep [14]. Zhang et al. designed a multiview image acquisition device by adopting the T2T-ViT method and introducing the SE attention mechanism, the LayerScale module, and ArcFace loss function. The improved T2T-ViT model achieves a recognition accuracy of 95.9% and an F1-Score of 95.5%. The ViT model is a heavyweight model with a huge number of parameters, making it difficult to meet lightweight requirements [15].

In addition, some scholars have conducted research based on the Siamese network. Zhang et al. constructed two datasets and designed RF_Block and EI_Block. They introduced a 3D attention mechanism to construct SAM_Block; the accuracies on 100 little-tailed cold sheep datasets were 97.2% and 90.5%, and the accuracies on small sample training were 92.1% and 86.5%, respectively. However, the size of the model is 112.2 MB, and the average recognition time is 49.7 ms [16].

Some scholars have also conducted research on model lightweighting. Zhang et al. lighten the model based on the YOLOv5s model. On the self-constructed dataset containing 63 small-tailed frigid sheep, the parameters and computational complexity of LSR-YOLO is 4.8 M and 12.3 GFLOPs, which are 25.5% and 33.4% lower than that of YOLOv5s, respectively, and the model size is only 9.5 MB when the mAP@50 reaches 97.8%, but there is still some room for compression in the number of model parameters and FLOPs [17]. Zhang et al. made improvements based on the YOLOv7-tiny module. Finally, they obtained the YOLOv7-SFR algorithm through knowledge distillation, with the number of parameters, model size, and average recognition time of 5.9 M, 11.3 MB, and 3.6 ms, respectively, on the self-constructed sheep face dataset; mAP@50 was 96.9%. However, with the introduction of the Dyhead module, the parameter and model sizes increased by 3.3 M and 6.5 MB, respectively [18].

The current research on lightweight sheep face recognition faces four core challenges: the number of model parameters and computational volume are too large, the number of parameters and FLOPs of traditional CNNs (e.g, ResNet50V2, YOLOv4) and Transformer architectures (e.g, ViT) are far more than the upper limit of the deployment of the mobile terminal, and the attention modules, such as CBAM, ARM, and so on, have introduced additional parameter overheads while improving the accuracy; Complex scene robustness is insufficient, and shallow networks (e.g, AlexNet) are poorly adapted to occluded scenes; it is difficult to balance lightweighting and accuracy, and although existing methods (e.g, LSR-YOLO, YOLOv7-SFR) compress parameters, the FLOPs and model size still affect the ultimate lightweighting, so it is necessary to optimize the parameter and computation volume in depth while guaranteeing the accuracy.

Facing these challenges, in this paper, the PFL-YOLO model is proposed, which aims to fill the gap in lightweight sheep face recognition and provide a new solution. The model is lightweight based on YOLOv8n, and by optimizing the model structure and parameter configurations, the number of parameters and computational complexity are significantly reduced to make it more adaptable to mobile devices or devices with limited computational resources while ensuring higher recognition accuracy. The main contributions of this study include the following:

- In this study, a dataset of sheep facial images was constructed through multi-angle image acquisition of 60 Australian and Lake hybrid sheep (bred from Australian White Sheep and Hu Sheep) in the Linxia Hui Autonomous Prefecture, Gansu Province, China.
- To meet the requirements of lightweight sheep face recognition, this study proposes the PFL-YOLO model. The EHConv module is embedded in the backbone network, which greatly reduces the number of parameters and computations while maintaining the receptive field and enhances the ability to extract local fine features such as wool texture and horn shape. The RC2f module is designed to realize the efficient interaction between shallow detail features and deep semantic features through the residual ladder network structure. The ESPPF module is proposed to fuse features of different scales and enhance the semantic information of the sheep face feature map through adaptive spatial pyramid pooling. The PFDetect module is constructed, which greatly reduces the number of parameters and the computation of the detection head through cross-layer parameter sharing and an attention mechanism.
- The PFL-YOLO model maintains an extremely low level of parameter number, computational complexity, and model size while being able to recognize a single sheep face efficiently and accurately. In addition, the PFL-YOLO model is superior to mainstream object recognition models in recognizing the positive features of sheep faces and is suitable for use in mobile devices, embedded devices, and surveillance systems.

## 2. Materials and Methods

### 2.1. Sheep Face Image Dataset

#### 2.1.1. Image Acquisition

The sheep facial image dataset of this study was obtained from the Luyuanxin Meat Sheep Industrial Park, Linxia County, Linxia Hui Autonomous Prefecture, Gansu Province, China. In July 2024, the researchers used a Redmi Note 12 Turbo mobile phone to capture facial videos of 60 healthy 4- to 5-month-old Australian–Hu crossbred sheep at 1920 × 1080 video resolution and 60 frames per second. The Australian–Hu crossbred sheep is a crossbreed between the Australian White Sheep and the Hu Sheep, and their faces have no or short horns, long ears, precise contours, light skin color, and gentle expressions. In the data acquisition process, due to the shy character of the Australian–Hu crossbred sheep and their remarkable habit of living in groups, they will often gather and move in an unorganized manner in the natural state, which will lead to the face of the sheep to be captured being obscured by the other sheep, which will affect the data annotation in the later stage of the study. Therefore, this study adopts the method of fixing the sheep to capture the images.

Three staff members in the sheep house, Z, R, and L, performed the image acquisition task for ease of presentation. During the specific operation, Z quickly stood at the position of the sheep’s front legs and precisely fixed the sheep’s neck to ensure the relative stability of the sheep’s head. At the same time, R took a position at the sheep’s hind legs and worked closely with Z to stabilize the sheep’s body. L held the Redmi Note 12 Turbo phone at a distance from the sheep. Holding the Redmi Note 12 Turbo phone, L photographed the sheep’s face from a distance of 0.25m to 1.0m, with a full angle range of 0° to 180° horizontally and a multi-angle range of 45° to 135° vertically, and the duration of the shot was controlled to be within 1 to 2 min. After a sheep is photographed, R airbrushed the tail of the sheep to avoid repeated shots.

#### 2.1.2. Image Preprocessing

**Figure 1:**
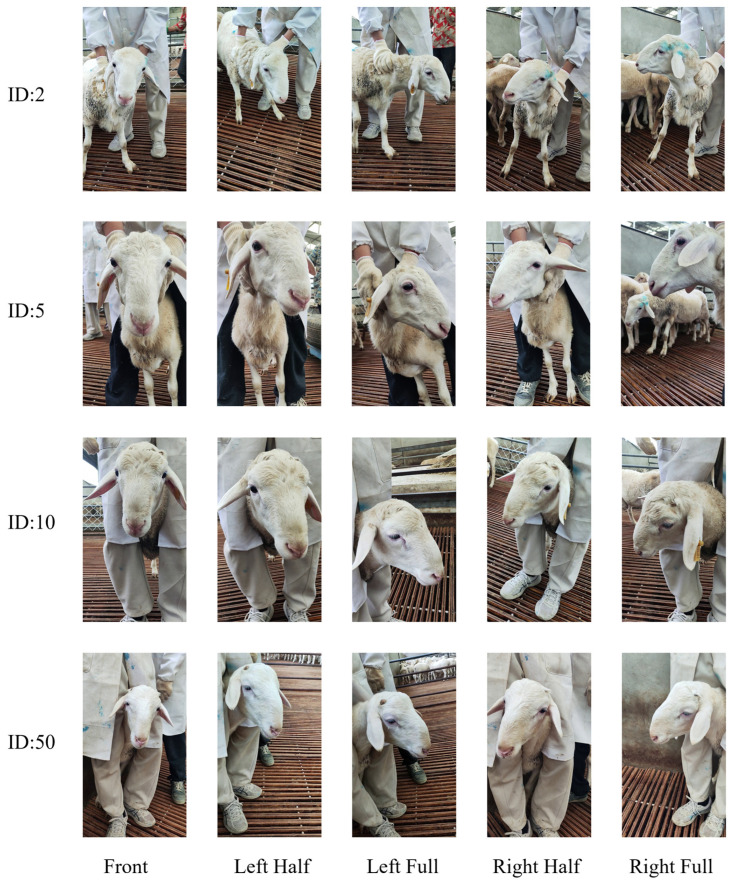
Images of sheep’s faces at different angles. Five views with IDs of 2, 5, 10, and 50 for four sheep: front view, left half view, left view, right half view, and right view.

In data processing, the Structural Similarity Index Measure (SSIM) [19] is used as a key index to measure the similarity of two images, and a value of SSIM close to 1 indicates that the two images are highly similar. The process of data processing was as follows: firstly, frames were extracted from the captured sheep face video at an interval of 30 frames to prevent high similarity due to the small frame interval, and a total of 10,225 images with a resolution of 1920 × 1080 were obtained; secondly, in the manual filtering stage, the images that did not contain the sheep’s face, the blurred images of the sheep’s face, and images that lacked the facial features due to high noise and exposure were filtered; and finally, SSIM was utilized to evaluate the similarity of the screened images in each category, and the set SSIM threshold was 0.75 to exclude images with high similarity. After manual and similarity screening by SSIM, 6418 high-quality sheep face images were finally obtained. The sheep face images from different angles are shown in Figure 1. The SSIM calculation formula is defined as follows:

(1) $$SSIM\left(x,y\right)=\frac{\left(2\mu_{x}\mu_{y}+C_{1}\right)+\left(2\sigma_{xy}+C_{2}\right)}{\left(\mu_{x}^{2}+\mu_{y}^{2}+C_{1}\right)+\left(\sigma_{x}^{2}{+\sigma }_{y}^{2}+C_{2}\right)}$$

where *x* and *y* denote two images, respectively, and *μ_x_* and *μ_y_* are the mean values of images *x* and *y* and are the variances of images *x* and *y*, respectively. *σ_xy_* is the covariance of images *x* and *y*. *C*_1_ = (k_1_L)^2^, *C*_2_ = (k_2_L)^2^ are constants set to avoid the denominator from being zero, L is the dynamic range of pixel values (usually 255), and k_1_ and k_2_ are microscopic constants that typically take values of 0.01 and 0.03.

#### 2.1.3. Data Annotations

**Figure 2:**
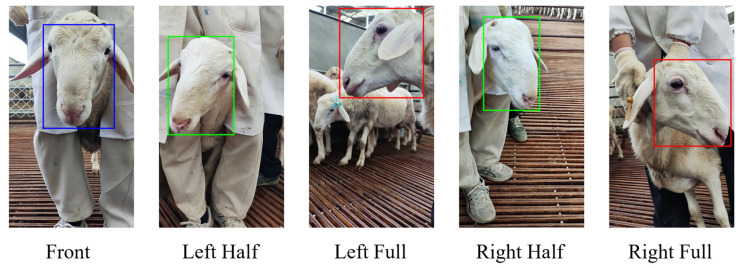
Schematic labeling of different angles of the sheep’s face.

In the data annotation process, this paper chose to use Make Sense, a free and open-source online annotation tool, to annotate 6418 sheep face images. In the annotation process, the rectangular box was used for labeling, and the labeled area was from 2 cm above the sheep’s forehead to below the sheep’s nose; in addition, it was necessary to ensure that the labeled area did not contain the sheep’s ear markers to reduce the impact of the sheep’s ear markers on sheep face recognition. After the labeling was completed, the results were exported to a .txt file. The contents of the .txt file included the ID, the x-coordinate of the center point of the bounding box, the y-coordinate of the center point, the width, and the height; to ensure that the labeling results were rigorous, all the contents of the labeling file were verified. After rigorous manual and SSIM similarity screening, each image in the dataset contained only one valid annotated target (GT). Figure 2 shows the annotation schematic of different angles of the sheep’s face.

#### 2.1.4. Data Enhancement

To enhance the robustness and generalization of the dataset, this study used the Albumentations library to augment the sheep face dataset to simulate the facial condition of sheep in different scenes. The six enhancement methods of horizontal and vertical flip, random 90-degree rotation, panning zoom and rotation, random brightness and contrast adjustment, and hue saturation and luminance adjustment were firstly selected to adapt to the diversified scene variations, such as different angles and light intensities, etc. In data enhancement, one of these six enhancement methods was randomly selected each time to enhance the image; this random selection strategy not only improved the generalization ability of the dataset but also avoided over-enhancement of the dataset. After data enhancement, the number of images in the dataset was expanded to 12,836. Subsequently, it was divided into a training set (10,272 images), a validation set (1282 images), and a test set (1282 images) according to the ratio of 8:1:1 for subsequent model training, validation, and performance evaluation. After completing the data enhancement operation, the labeled files after data enhancement were verified, focusing on checking whether the coordinates of the center point, as well as the width and height, were out of the boundary range. Table 1 shows the divided dataset. Table 2 shows the number of images of 60 sheep before and after data augmentation.

### 2.2. PFL-YOLO Model

**Figure 3:**
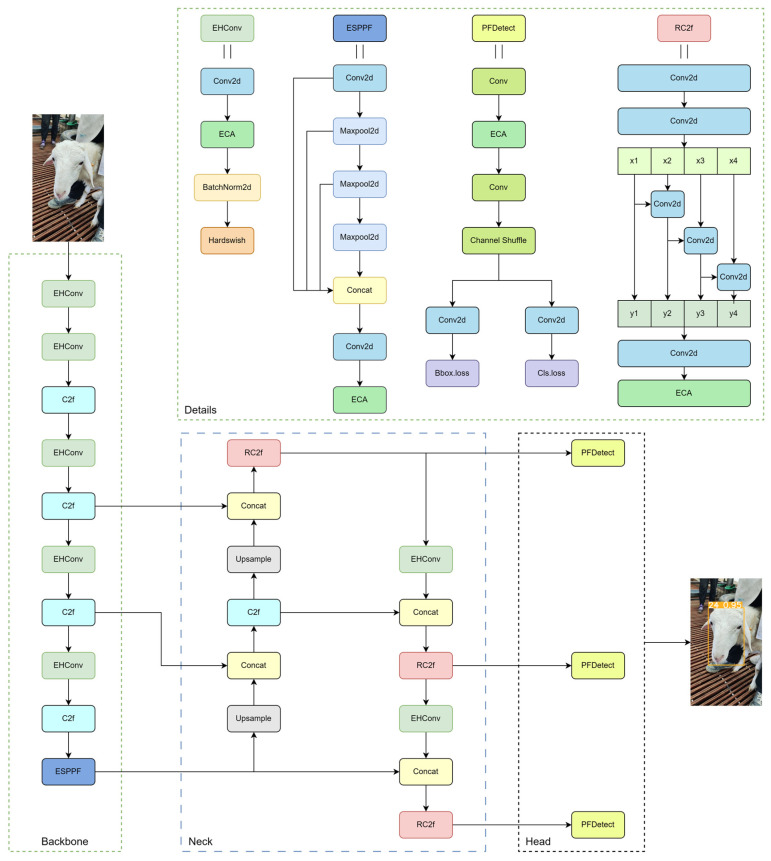
The architecture of PFL-YOLO network covers the backbone, neck, and head parts, and it improves four modules: EHConv, ESPPF, RC2f, and PFDetect. The Details section of the figure further shows the specific structure of each improved module, and each Conv2d function adopts grouped convolutional design. In addition, the ECA attention mechanism is introduced in the architecture, and the Hardswish activation function is applied.

YOLOv8n, the lightest model in the YOLOv8 series, is widely used in lightweight recognition scenarios. On the self-built sheep face dataset, YOLOv8n achieved high recognition accuracy and had a relatively low demand for computational resources. However, in real production environments, mobile and embedded devices may face computational resource limitations, affecting the model’s recognition accuracy and detection speed. Based on this, this study lightened and improved the YOLOv8n model to reduce its dependence on computational resources, and in this paper, the improved model was named PFL-YOLO. Its network structure diagram is shown in Figure 3.

#### 2.2.1. ECA Attention Mechanism

**Figure 4:**
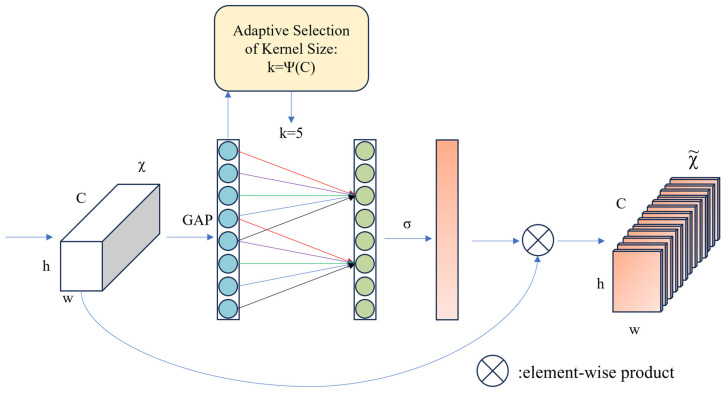
The network structure of the ECA attention mechanism.

ECA (Efficient Channel Attention) [20] is a channel attention mechanism that enables the model to focus on key parts of the input data, such as target regions in the labeled data. It adaptively weights the features in the channel dimension, prompting the model to pay more attention to information-rich channels while suppressing relatively unimportant channels, thus improving the feature representation capability of the model. Compared with the traditional attention mechanism, the ECA module avoids the complex dimensionality reduction and upgrading process. It has the characteristics of high efficiency and lightweight qualities, which is very suitable for the task of a lightweight model. The ECA module first obtains the global features through Global Average Pooling and then uses the Conv1D and Sigmoid activation function to compute the convolution kernel by using the formula to compute the convolutional kernel, then adjusts the shape of the output of the convolutional layer to generate the weights of each channel. Subsequently, each channel is multiplied by the weight of the corresponding channel, making the network more focused on the features that are favorable for the current task. The ECA module diagram is shown in Figure 4:

#### 2.2.2. Hardswish Activation Function

**Figure 5:**
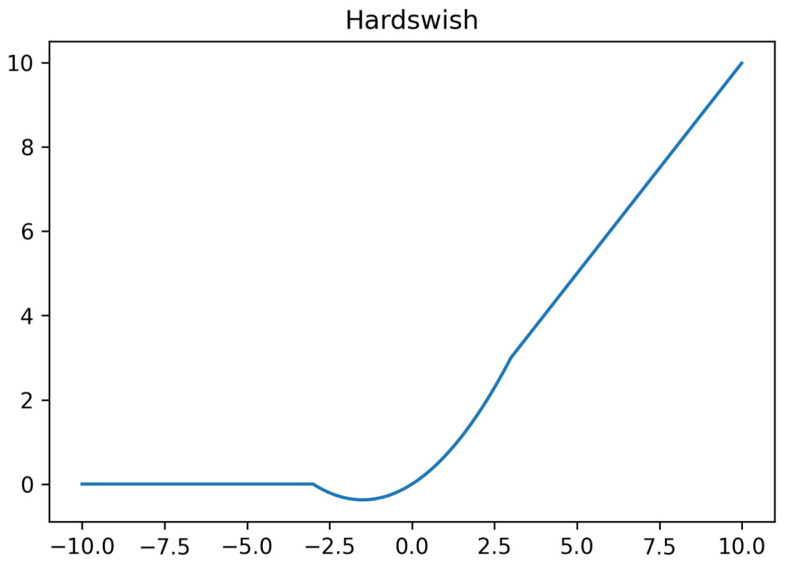
The graph of the Hardswish activation function.

Hardswish is an activation function optimized for mobile devices and embedded systems. It dramatically improves computational efficiency by simplifying the form of the Swish function while maintaining its nonlinear properties. This is especially important for resource-constrained devices, which can speed up the model without sacrificing too much performance., Figure 5 shows the image of the Hardswish activation function. The definition of the Hardswish activation function is as follows:

(2) $$Hardswish\left(x\right)=\left\{\begin{array}{c} 0\;\;\;\;\;\;\;\;\;\;\;\;\;\;\;\;\;\;\;\;\;\;\;\;\mathrm{if}\;x\leq -3,\\ x\;\;\;\;\;\;\;\;\;\;\;\;\;\;\;\;\;\;\;\;\;\;\;\;\mathrm{if}\;x\geq +3,\\ x\cdot (x+3)/6\;\;\;\;otherwise \end{array}\right.$$

#### 2.2.3. Improved EHConv Module

**Figure 6:**
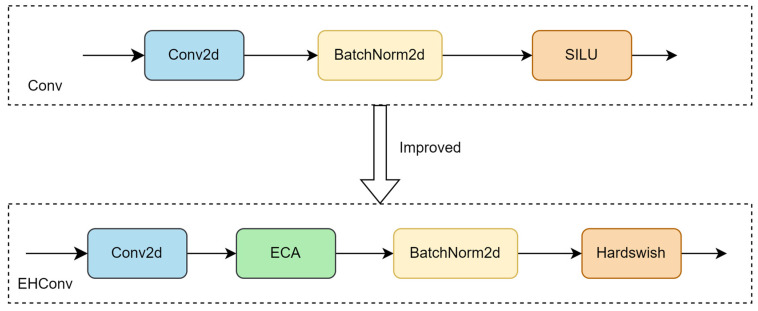
A comparison of the Conv module before and after improvement.

To strengthen the model’s ability to extract the basic features of the sheep face and capture the image details, improvements were made based on the Conv module to obtain the EHConv module. Specifically, the grouping operation is utilized in the Conv2d function, effectively maintaining the feature extraction ability while reducing the model complexity. Subsequently, the ECA attention mechanism is added after the Conv2d function, which focuses on the feature channels important to the sheep face recognition model. It thus improves the discriminative and expressive ability of the features. BatchNorm2d is used to accelerate the training process and improve the training efficiency of the model. Finally, the Hardswish activation function is used, which can realize more efficient nonlinear transformation in some cases and help the model to learn more complex features. The comparison diagram of the Conv module before and after the improvement is shown in Figure 6:

#### 2.2.4. Improved RC2f Module

**Figure 7:**
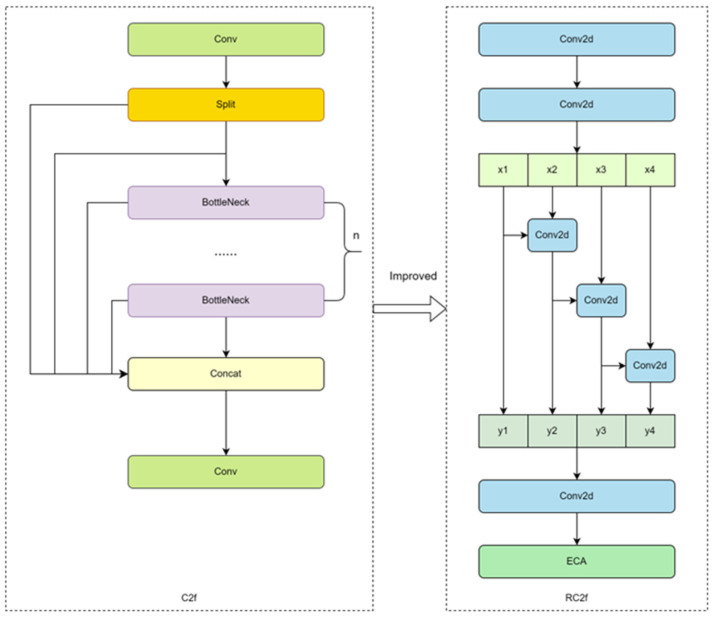
Comparison of C2f module before and after improvement.

Aiming at the challenges of performance and efficiency of the C2f module of YOLOv8 on resource-constrained devices, an improved scheme, namely the RC2f module, was proposed. The design of the RC2f module refers to the network module structure of Res2Net [21], and by reconfiguring the structure, more fine-grained processing of feature information is realized. In the RC2f module, the initial feature extraction is first carried out through two Conv2d layers and then divided into four groups regarding channel dimensions. Starting from the second group [22], each group of features handles its information independently, receives and integrates the channel information of the previous group, and realizes cross-group fusion of the features through the summing operation. This design enables the model to capture complex feature relationships more effectively. The fused features are spliced in the channel dimension, and a Conv2d layer further refines the features. Finally, a lightweight ECA attention mechanism is introduced to adaptively adjust the channel weights, which enhances the model’s ability to focus on key features while maintaining computational efficiency. Through these improvements, the unnecessary computational cost was cut down, and the computational efficiency was improved. Also, in scenarios such as sheep face recognition, the feature information can be processed more finely, which is more conducive to focusing on the key parts of the sheep’s face. The structure diagrams before and after the improvement of the C2f module are shown in Figure 7:

#### 2.2.5. Improved ESPPF Module

**Figure 8:**
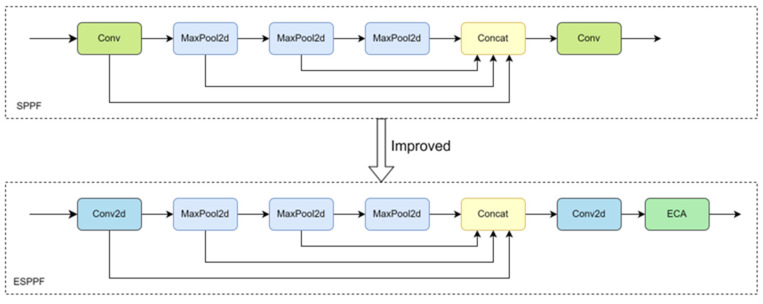
A comparison of the SPPF module before and after improvement.

In order to realize multi-scale feature fusion with fewer parameters and thus enhance the semantic information of the sheep face feature map, the ESPPF module based on the SPPF module was proposed. Specifically, the first Conv module in SPPF is firstly replaced by the Conv2d function with grouping operation, which can effectively reduce the number of parameters. Subsequently, after a series of pooling operations, the information on the sheep’s facial features on different scales is fused in the channel dimension. Then, the Conv2d function with grouping operation is applied again to reduce the number of parameters and computational complexity. Finally, the ECA attention mechanism filters the input channel information to optimize the feature fusion effect. The comparison diagram of the SPPF module before and after the improvement is shown in Figure 8:

#### 2.2.6. Improved PFDetect Detection Header

In this study, the original Detect detection head was optimized, and the PFDetect detection head was proposed to achieve the lightweighting of the YOLOv8n model for the sheep face recognition task. The core improvement of PFDetect lies in merging the structure by streamlining the number of parameters and effectively reducing computational complexity. First, two independent branches exist in the original Detect detector head, which increases the number of parameters in the model and raises the computational burden. For this reason, merging the branches was adopted to reduce the number of parameters by sharing the Conv module. This improvement simplifies the model structure and increases the efficiency of the model operation by sharing the feature extraction process. Second, to further reduce the number of parameters, grouping operations are used in the merged Conv module, which effectively reduces the number of parameters and computation. However, grouping convolution may limit the information exchange between groups and affect the expressiveness of features. For this reason, Channel Shuffle [23] was used, a technique that effectively improves the ability of channel information exchange between different groups and ensures feature richness. In addition, to enhance the model’s ability to capture key features, an ECA attention mechanism was added between the two Conv modules, which can adaptively focus on important feature channels, thus improving the model’s sensitivity to key information and enhancing the feature discrimination ability.

**Figure 9:**
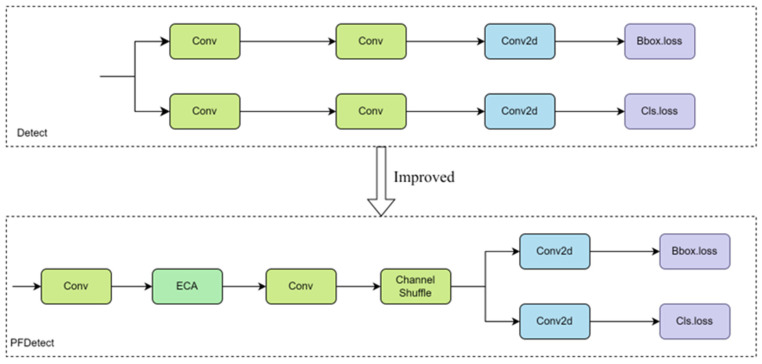
Comparison of Detect module before and after improvement.

These improvements significantly reduce the number of parameters and computational complexity of the model, improve the operational efficiency of the model, and enhance its applicability on resource-constrained devices, providing strong technical support for real-time application scenarios such as sheep face recognition. The structure of Detect before and after the improvement is shown in Figure 9:

### 2.3. Experimental Configuration and Parameter Setting

Given the large size of the sheep face dataset in this study and the high resolution of 1920×1080, this can lead to excessive training time. In order to reduce the training time and improve efficiency, server was used throughout this experiment. The operating system of the server was CentOS 8.5; the CPU was Intel(R) Xeon(R) Gold 6148 CPU @ 2.40GHz (Intel Corporation, Santa Clara, CA, USA), and the GPU was NVIDIA Tesla V100(NVIDIA Corporation, Santa Clara, CA, USA). The specific experimental environment settings are shown in Table 3. All models were trained using a single card and without pretrained weights. The training hyperparameter settings are shown in Table 4.

### 2.4. Evaluation Indicators

In order to evaluate the accuracy of the PFL-YOLO model for recognizing sheep’s faces, precision, recall, mAP@50, mAP@50:95, and F1-Score were selected as the accuracy evaluation indices in this study. The number of parameters, floating point operations per second, model size, average detection time (Dt), and frame rate (FPS) were selected as lightweight indicators for the model. The formulas for calculating precision, recall, F1-Score, mAP (mean average precision), average detection time (Dt), and FPS are defined as follows:

(3) $$Precision=\frac{TP}{TP+FP}$$

(4) $$Recall=\frac{TP}{TP+FN}$$

(5) $$F1-Score=\frac{2\;*\;Precision\;*\;Recall}{Precision+Recall}$$

(6) $$mAP=\frac{\sum_{1}^{N}\;\int_{0}^{1}\;P\left(R\right)dR}{N}$$

(7) $$D_{t}=\frac{1}{M}\sum_{i=1}^{M}\;t_{i}$$

(8) $$FPS=\frac{1}{D_{t}}$$

where *TP* (True Positive) means that the model accurately recognizes the sheep IDs; *FP* (False Positive) indicates that the model incorrectly recognizes a sheep ID as another sheep ID; and *FN* (False Negative) means that no sheep IDs are recognized even if the corresponding IDs exist. In Equation (6), *N* represents the number of categories in the sheep face dataset, which is 60 in this dataset. Ap is the area under the P-R curve, i.e., *mAP* denotes the average AP of the different categories. The detection time *t* is the sum of pre_process, inference, and post_process, and *M* is the number of images in the test set.

## 3. Results

### 3.1. Results and Analysis of Ablation Experiments

In order to understand the impact of the improved module on the model, ablation experiments were designed in this study. The ablation experiments were conducted with the same server configuration and the same hyperparameters. The results of the ablation experiments are shown in Table 5 and Table 6, where √ indicates the use of the module and is left blank if it is not used.

The introduction of the ESPPF module into the YOLOv8n model alone reduced the number of parameters by 5.3% (0.16 M) but resulted in a 0.7% decrease in recall and a 0.2% decrease in mAP@50; this suggests that the ESPPF module has a role in lightweighting the model but has an impact on the model’s detection performance, especially on the recall metrics.

On the basis of YOLOv8n+ESPPF, the introduction of the EHConv module alone can increase the precision by 0.1%, reduce the number of parameters by 23.8% (0.72 M), and compress the FLOPs by 14.8% (1.2 G), but it can lead to a decrease in the recall rate by 0.2%. This suggests that the EHConv module excels in improving model accuracy and lightweighting, with relatively little impact on recall. The introduction of the RC2f module alone reduces the number of parameters by 24.8% (0.75 M), FLOPs by 13.5% (1.1 G), and model size by 25% (1.5 MB), but it results in a 0.2% decrease in the recall rate and a 0.4% decrease in mAP@50:95. While the RC2f module has a significant effect in terms of model compression, it has an acceptable impact on detection performance. The introduction of the PFDetect module alone can increase precision by 0.1%, reduce the number of parameters by 28.4% (0.86 M), FLOPs by 33.3% (2.7 G), and model size by 28.3% (1.7 MB) while decreasing mAP@50:95 by 0.4%. This suggests that the PFDetect module effectively improves accuracy while compressing the model with relatively little effect on mAP@50:95.

Based on the YOLOv8n+ESPPF model, the EHConv, RC2f, and PFDetect modules were combined in pairs to explore the influence of the combination effect of these modules on the model. When EHConv is combined with the RC2f module, the mAP@50:95 is reduced by 0.9%, but the parameters are reduced by 43.6% (1.31 M), the model size is reduced by 41.2% (2.6 M), and the FLOPs are reduced by 27.1% (2.2 G). Recall is improved by 0.2%. When EHConv is combined with the PFDetect module, the recall rate is reduced by 0.5%, but the number of parameters is reduced by 47% (1.42 M), the model size is reduced by 45% (2.7 MB), and the FLOPs are reduced by 46.9% (3.8 G). When RC2f is combined with the PFDetect module, the recall rate is reduced by 1.1%, but the number of parameters is reduced by 48% (1.45 M), the model size is reduced by 46.7% (2.8 MB), and FLOPs are reduced by 45.6% (3.7 G).

After the integration of the four modules, the number of model parameters was compressed to 33.4% (1.01 M) of the original size, and the FLOPs were reduced by 59.3% (4.8 G); the core detection accuracy (mAP@50 99.5%) was maintained, but the mAP@50:95 index decreased by 1.0%. The results show that the combination of the four modules achieves an effective balance between model lightness (model parameter number 1.01 M, model size 2.1 MB) and high accuracy.

### 3.2. Comparison Experiment

In this comparison experiment, to comprehensively evaluate the model performance, several lightweight versions of the YOLO series are selected for comparison. Specifically, these include YOLOv5n, YOLOv7-tiny [24], YOLOv8n, YOLOv9-t [25], and YOLO11n, the most lightweight models within their respective series. In addition to investigating the effect of the lightweight model further, this study carries out a series of innovative lightweight improvements based on YOLOv8n. The backbone layer of YOLOv8n was modified with the help of Hugging Face’s timm library. Specifically, several layers before the SPPF module were replaced with a more lightweight model architecture, which includes the mobilenetv3_small_050, mobilenetv4_conv_small_035, ghostnet_050, and lcnet_035 models. The interpretation of the above models is as follows: mobilenetv3_small_050 adopts the miniature model of MobileNetV3 [26] with a model width of 0.5; mobilenetv4_conv_small_035 selects the small convolutional model of MobileNetV4 [27], a small convolutional model with the same model width of 0.5; ghostnet_050 uses the GhostNet [28] model with the model width kept at 0.5; and lcnet_035 employs the LCNet [29] model, which also has a model width of 0.5. In this paper, the transformed models are named V3-YOLOv8n, V4-YOLOv8n, LCNet-YOLOv8n, and Ghost-YOLOv8n for the subsequent analysis and comparison of model performance.

#### 3.2.1. PFL-YOLO: Optimal Balance of Performance and Efficiency

In a comprehensive analysis of the performance of various YOLO series models and their improved versions, the PFL-YOLO model demonstrated distinct advantages. First, in terms of key performance metrics, the results are shown in Table 7. PFL-YOLO achieved 98.5% precision, 98.8% recall, 99.5% mAP@50, 87.4% mAP@50:95, and 98.65% F1-Score. These results indicate that although PFL-YOLO did not achieve the highest score across all evaluation criteria, it maintained a consistently competitive level across all major performance indicators, particularly excelling in mAP@50 and F1-Score, which reflects an excellent balance between detection accuracy and efficiency. From the perspective of model performance efficiency, the results are shown in Table 8. The PFL-YOLO model requires only 1.01 M parameters, 3.3 GFLOPs of computational complexity, and a model size of 2.1 MB to achieve an average detection time of 11.2 ms and a detection speed of approximately 89.4 images per second. Compared to other models such as YOLOv5n and YOLOv7-tiny, PFL-YOLO significantly reduces computational resource requirements and model size while maintaining similar or even superior detection performance, making it especially suitable for deployment on resource-constrained devices.

Further comparison shows that although some models, such as YOLOv9-t, are slightly higher than PFL-YOLO in terms of precision and recall, they tend to require more parameters, higher computational complexity, and larger model sizes, e.g., YOLOv9-t has 3.51 million parameters and 15.3 GFLOPs, which results in a much slower inference than PFL-YOLO. In addition, the modified backbone networks like V3-YOLOv8n and Ghost-YOLOv8n, although similar to PFL-YOLO in terms of the number of parameters and model size, are inferior to PFL-YOLO in terms of detection performance metrics, which once again demonstrates that PFL-YOLO does a particularly good job of optimizing the balance between model efficiency and performance.

In summary, PFL-YOLO not only excels in detection performance but also demonstrates significant advantages in computational efficiency and model size. This high performance and low resource consumption make PFL-YOLO particularly suitable for applications in scenarios with high real-time and computational resource requirements, such as target detection tasks in mobile devices or edge computing environments.

#### 3.2.2. Balance of High Precision and Low Parameters

**Figure 10:**
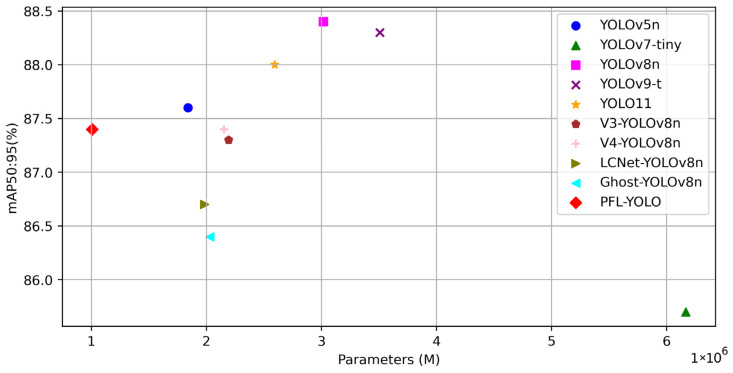
Scatter plot of accuracy and number of parameters.

The PFL-YOLO model substantially reduces the number of parameters and complexity while maintaining high accuracy, reducing the demand for computational resources; it can be deployed on resource-constrained devices to recognize sheep faces in different scenarios effectively, and it provides a highly efficient and lightweight solution for sheep individual recognition. Figure 10 shows the relationship between the accuracy of the ten target detection models and the number of parameters, from which it can be seen that the closer to the point in the upper left corner, it proves that the model has a high recognition accuracy while having a low number of parameters. The mAP@50:95 accuracy of the PFL-YOLO model is 87.4%; among the other target detection models, the index of YOLOv8n is the highest, amounting to 88.4%, and compared with YOLOv8n, the PFL-YOLO model loses only 1%, but the number of parameters is reduced by 2M. The PFL-YOLO model achieves a balance between high accuracy and lightweight.

#### 3.2.3. Comparison of Accuracy and Loss Curves for Different Models

**Figure 11:**
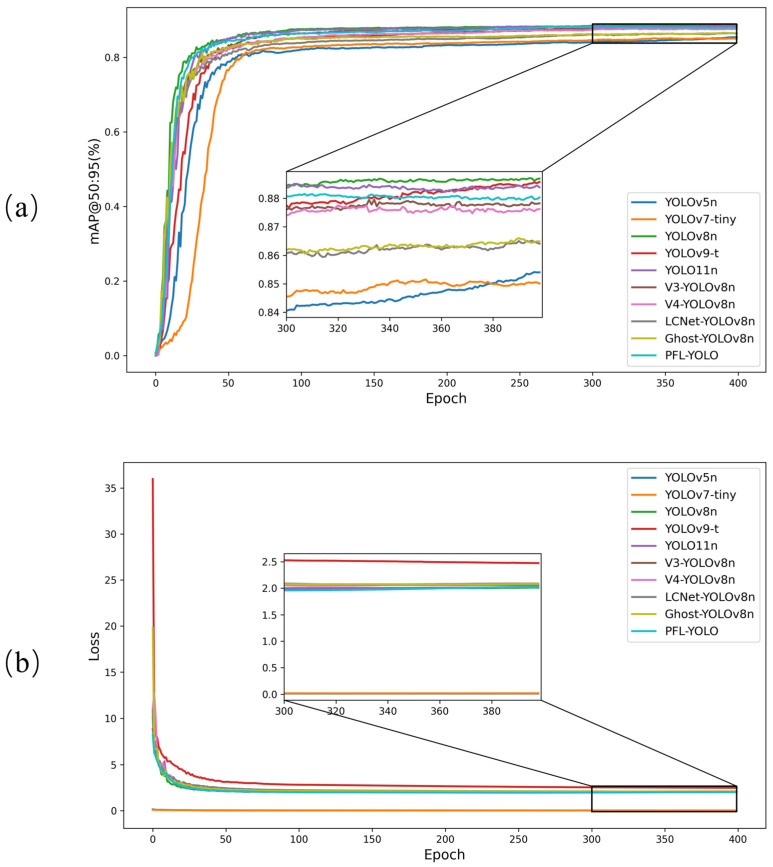
Comparison of model accuracy and loss curves. (**a**) mAP@50:95 curve graphs of different models. (**b**) Validation loss curves of different models.

Figure 11 shows the mAP@50:95 accuracy curves and the validation set loss curves of the ten object detection models in the validation set. In Figure 11a, the mAP@50:95 of PFL-YOLO is slightly lower than that of YOLOv8n and YOLOv9-t but better than that of other models with modified backbones. The lower accuracy of YOLOv5n and YOLOv7-tiny indicates their limitations in complex tasks. In the later training phase (300–400 epochs), mAP@50:95 is basically stable for all models. In Figure 11b, the loss values of all models decrease with an increasing number of training epochs, indicating that the models gradually converge during the training process. In addition, the loss of most models tends to be stable around 100 epochs. The loss of PFL-YOLO fluctuates slightly around 300 epochs but eventually stabilizes around 380–400 epochs.

#### 3.2.4. Comparison of Parameter Counts and FLOPs for Models

**Figure 12:**
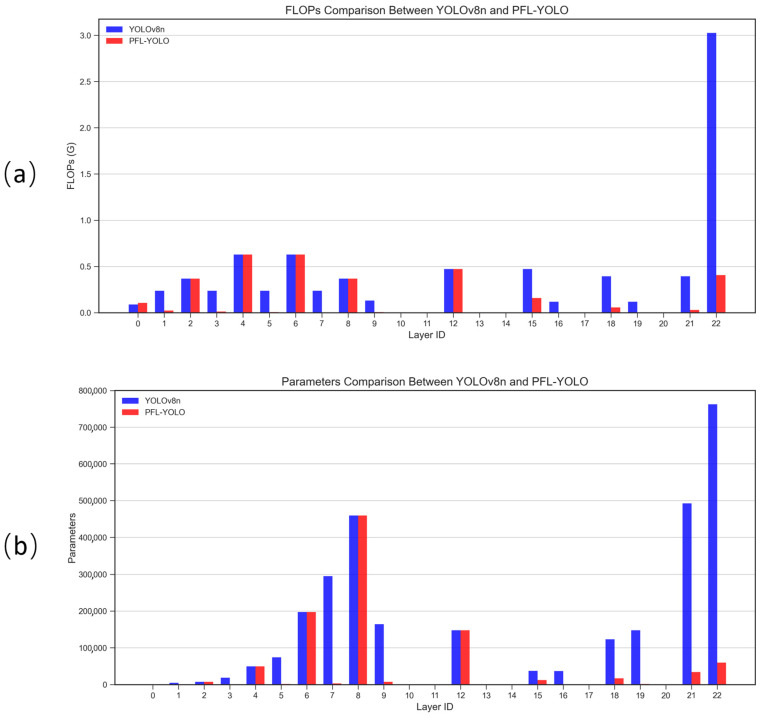
A comparison of the number of parameters and FLOPs between YOLOv8n and PFL-YOLO. (a) A comparison of FLOPs at each layer between YOLOv8n and PFL-YOLO. (b) A comparison of the number of parameters in each layer between YOLOv8n and PFL-YOLO.

Figure 12 illustrates the histogram comparison of the number of parameters and FLOPs for the YOLOv8n and PFL-YOLO models. In Figure 12a, the FLOPs of PFL-YOLO in most layers are significantly lower than those of YOLOv8n, especially in the 22nd layer, where the FLOPs of YOLOv8n is 3.025 G, while PFL-YOLO is only 0.405 G, indicating that PFL-YOLO has more advantages in computational efficiency. In Figure 12b, the number of parameters of PFL-YOLO is significantly lower than that of YOLOv8n in the 22nd layer, where the number of parameters of YOLOv8n is 762,244 while that of PFL-YOLO is only 59,861, which further proves the lightweight advantage of PFL-YOLO.

PFL-YOLO outperforms YOLOv8n in terms of FLOPs and the number of parameters, which makes it more suitable for use in resource-constrained environments.

Table 9 shows the statistical results of the PFL-YOLO model with the lightweight detection model. The number of PFL-YOLO parameters is only 1.01 M (33.4% of YOLOv8n and 54.8% of YOLOv5n), the FLOPs are only 3.3 G (40.7% of YOLOv8n), and the model size is compressed to 2.1 MB (55.2% of YOLOv5n). Compared with YOLOv7-tiny, the number of parameters is reduced by 83.6%, and the computation amount is reduced by 75.5%, but it can still maintain high accuracy with very low resource consumption (mAP@50:95 87.4%). In terms of precision–efficiency balance, the PFL-YOLO model achieves a mAP@50 of 99.5% (equal to YOLOv9-t and YOLO11n), which is only 0.1% lower than YOLOv7-tiny (99.6%), and the mAP@50:95 (87.4%) is slightly worse than YOLOv8n (88.4%). However, it approaches the performance of mainstream models with 33.4% parameters and 40.7% computation, which is more suitable for resource-constrained scenarios.

#### 3.2.5. Comparison of Heatmap Effects of Different Models

Heatmaps can effectively visualize the region of concern of the algorithm through the change of color shades to intuitively present the model’s determination of the likelihood of the existence of different regions and targets in the image. The Gradient-weighted Class Activation Mapping (Gradient-CAM) [30] technique has been used in this study to visualize the effect of sheep face recognition.

**Figure 13:**
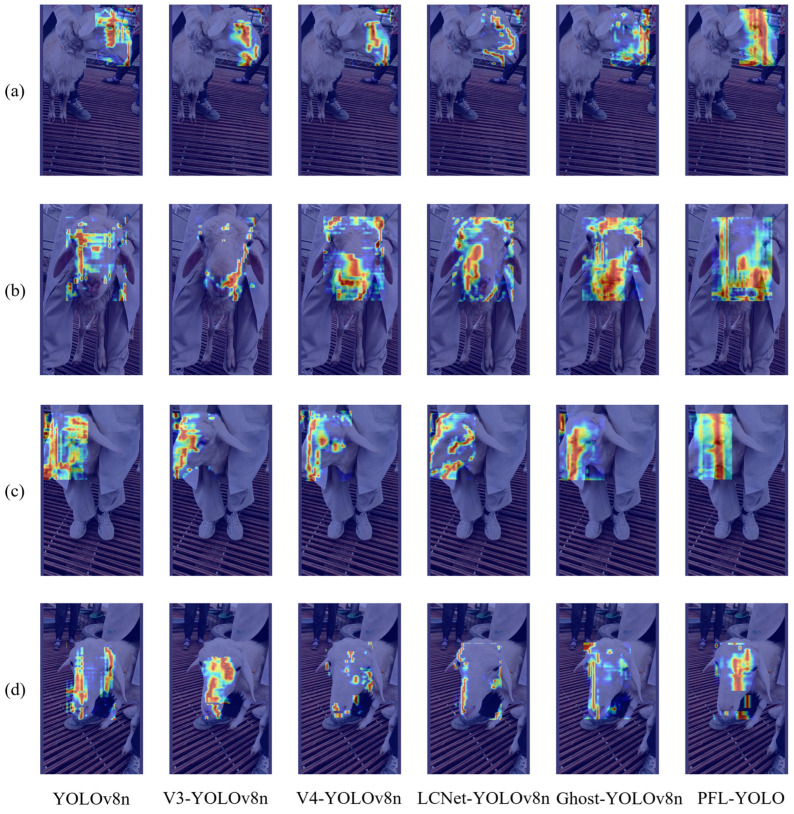
Comparison of heatmap visualization effects: (**a**) sheep face image with ID 4, (**b**) sheep face image with ID 6, (**c**) sheep face image with ID 11, and (**d**) sheep face image with ID 24. In the heat map, red indicates that the model pays more attention to the area, while blue represents less attention.

As shown in Figure 13, in images (a) and (b), the heatmap area of the PFL-YOLO model was significantly superior to that of YOLOv8n and other lightweight models. It shows that it can capture the key information of the image more comprehensively, especially when dealing with the frontal features of the sheep’s face, and can make full use of the large effective area to improve the recognition accuracy.

To deepen the comparison, the heatmap area of interest was calculated through a process involving grayscale conversion, binarization, contour extraction, and area computation of the contour-surrounded region. Table 10 lists the pixel area of the area of interest for the different models. In image (a), PFL-YOLO’s heatmap concern area (20,066 pixels) far exceeded that of other models. In image (b), its area reached 38,888.5 pixels, again surpassing others. In image (c), although PFL-YOLO’s 27,417.5 pixels were lower than YOLOv8n’s 29,417 pixels, it still outperformed other lightweight models. In image (d), its 13,686 pixels were lower than YOLOv8n (17,062.5 pixels) and V3-YOLOv8n (17,556 pixels) but higher than some lightweight models.

PFL-YOLO demonstrated a larger focus area across all images, indicating its ability to comprehensively capture key information during detection. In contrast, models such as YOLOv8n, V3-YOLOv8n, V4-YOLOv8n, LCNet-YOLOv8n, and Ghost-YOLOv8n exhibited smaller focus areas in certain images, suggesting incomplete capture of key areas in specific scenes.

#### 3.2.6. Comparison of Prediction Results of Different Models

**Figure 14:**
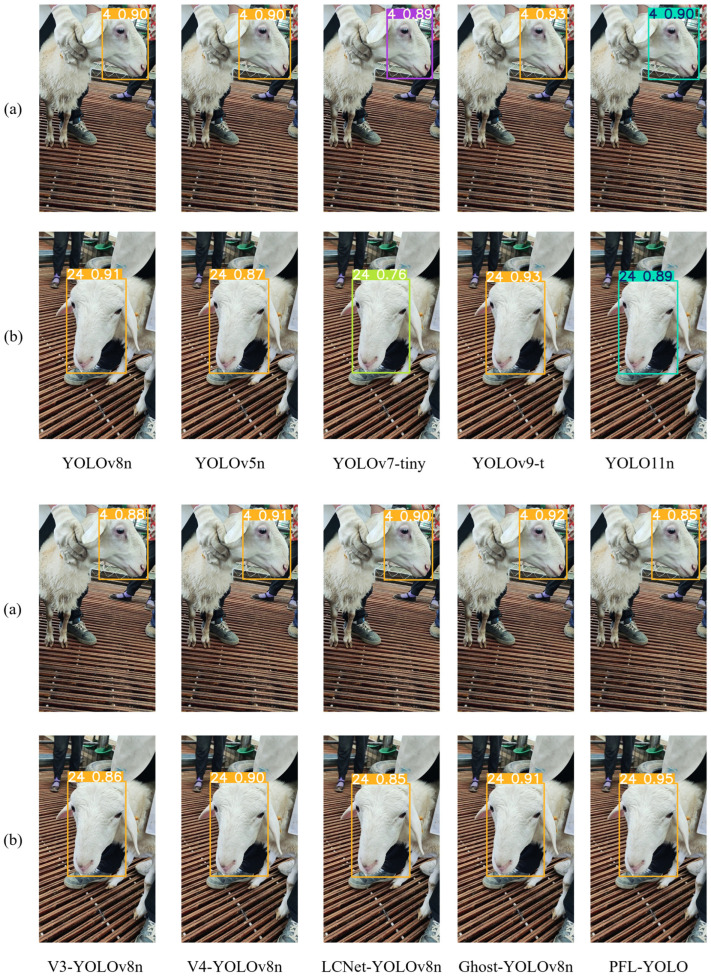
A comparison of the results of different models predicting sheep faces. (**a**) Sheep face image with ID 4 and (**b**) sheep face image with ID 24.

To evaluate the performance of different target detection models in the sheep individual recognition task, two images from the test set were randomly selected for sheep face recognition in this study. Figure 14 displays the prediction results of ten target detection models. In image (a), most models present relatively balanced recognition abilities, with prediction probabilities roughly centered at 90%. Notably, PFL-YOLO shows the lowest prediction probability of 85%, a 5% reduction compared to YOLOv8n, revealing its limitations in dealing with the lateral features of sheep faces, which impacts its generalization ability in this specific scenario. Conversely, in image (b), PFL-YOLO achieves the highest prediction probability of 95%, reflecting a 4% improvement over YOLOv8n. These findings highlight PFL-YOLO’s distinct advantage in capturing the frontal features of sheep faces, enabling robust recognition for sheep faces with prominent frontal characteristics. Although PFL-YOLO demonstrates suboptimal performance in some scenarios, its strengths in identifying the frontal features of sheep faces remain significant.

## 4. Discussion

Many scholars have carried out related research on lightweight sheep face recognition. For example, Zhang et al. based their work on the lightweight YOLOv5s model and achieved some results on the self-built dataset; the number of parameters, FLOPs, and model size of LSR-YOLO were reduced to a certain extent, and the mAP@50 also had good performance [17]. Zhang et al. based their work on the improved YOLOv7-tiny module. The obtained YOLOv7-SFR algorithm also shows good performance on the self-built sheep face dataset [18]. Compared with the sheep face recognition models proposed by these two scholars, the PFL-YOLO model in this study has greatly improved in terms of parameter quantity and mAP@50 indicators; the PFL-YOLO model maintains a higher mAP@50 (99.5%) while further reducing the number of parameters to 1.01 M and is also competitive in terms of average detection time and FPS. This indicates that the lightweight strategy in this study is more efficient in balancing model performance and resource consumption and can be better adapted to sheep face recognition in resource-constrained environments.

The data collection method in this study is manual, and this method is suitable for small farms. However, the manual collection method is time-consuming and labor-intensive for large farms. An automated acquisition scheme can be considered to solve this problem. Expressly, a 3m×3m fenced area can be set up on a large farm, and one sheep at a time can be guided into the area and allowed to move freely. Four video cameras are installed around the fence to take footage of the sheep. Image frames were extracted from the video at a frequency of 30 frames per second by video processing techniques. The extracted image frames are then processed using a sheep face detection model (which is only used to locate sheep faces and not to identify individuals) to filter out images containing sheep faces and assign a unique ID number to each sheep. This automated acquisition method effectively reduces the reliance on human resources, significantly improves work efficiency, and gives reuse value to the acquired data. Compared with the manual collection method, the automated scheme demonstrates higher efficiency and greater scalability, providing strong technical support for the intelligent management of large-scale farms.

The sheep face dataset constructed in this study contains only a single breed (Australian–Hu crossbred sheep) at this stage, and all the samples were collected in the sheep house environment. Although the robustness of the sheep face dataset has been improved by a series of data enhancement techniques, it lacks images of sheep faces from other breeds and complex scenes (high occlusion and low light), and we will work on expanding the dataset in the future to include images of sheep faces of multiple breeds and age groups in complex scenes to improve the generalization and generalization ability of the dataset. Robustness testing will be conducted on the expanded complex dataset to evaluate sheep face recognition capabilities, providing a new option for a high-precision, low-parameter general sheep face recognition model.

The mAP@50 and mAP@50:95 of PFL-YOLO on the self-built sheep face dataset are 99.5% and 87.4%, respectively, which is a 0.1% improvement over YOLOv8n on the mAP@50 metric, while the number of model parameters is compressed by 66.5%, and the computational complexity is reduced by 59.2%. It is worth noting that the average inference time of the model is 11.2 ms, which is mainly due to the extra computational overhead introduced by the bidirectional feature fusion structure of the RC2f module. Future research will focus on structured pruning and knowledge distillation, which can greatly reduce the number of parameters and FLOPs while maintaining accuracy. In addition, they can be applied in livestock breeding scenarios, accurately and stably play the individual identification function, and provide strong support for the intelligent management of the livestock industry.

## 5. Conclusions

In order to meet the lightweight requirement for sheep face recognition, the PFL-YOLO model is proposed in this study. The model achieves efficient feature processing through four innovative optimization strategies: the EHConv module is integrated into the backbone network to enhance image detail capture capability; the SPPF module is upgraded to the ESPPF module to expand the receptive field and improve semantic representation; and the RC2f module is employed to strengthen multi-scale feature fusion in the neck network. Additionally, the PFDetect module is introduced in the design of the detection head, where a parameter fusion strategy is applied to compress the number of parameters. The experimental results demonstrate that while maintaining a detection accuracy of 99.5% mAP@50, the model has only 1.01 M parameters (33.3% of YOLOv8n), with computational complexity reduced to 3.3 GFLOPs (40.8% of YOLOv8n). The model size is compressed to 2.1 MB (35% of YOLOv8n). This model exhibits significant advantages in the intelligent management of the sheep farming industry, enabling rapid and accurate identification of sheep and providing efficient solutions for functions such as precise feeding, disease monitoring, and behavior analysis.

## Figures and Tables

**Table 1:** The segmentation of the sheep face dataset.

	After Data Filtering	After Data Enhancement
Training Set	5136	10,272
Validation Set	641	1282
Test Set	641	1282
Total	6418	12,836

**Table 2:** The number of images per sheep before and after data augmentation.

ID	After Data Filtering	After Data Enhancement	ID	After Data Filtering	After Data Enhancement
0	134	268	30	109	218
1	73	146	31	137	274
2	80	160	32	98	196
3	78	156	33	148	296
4	78	156	34	101	202
5	76	152	35	119	238
6	68	136	36	117	234
7	85	170	37	120	240
8	109	218	38	156	312
9	87	174	39	122	244
10	117	234	40	97	194
11	62	124	41	147	294
12	100	200	42	120	240
13	62	124	43	108	216
14	110	220	44	120	240
15	120	240	45	164	328
16	107	214	46	98	196
17	67	134	47	101	202
18	112	224	48	127	254
19	97	194	49	156	312
20	79	158	50	126	252
21	117	234	51	106	212
22	68	136	52	168	336
23	90	180	53	131	262
24	86	172	54	129	258
25	101	202	55	119	238
26	76	152	56	55	110
27	132	264	57	113	226
28	122	244	58	114	228
29	90	180	59	109	218

**Table 3:** Hardware configuration and software environment for algorithm operation.

Hardware	Configure	Software	Version
CPU	Intel(R) Xeon(R) Gold 6148 CPU @ 2.40GHz	Operating System	CentOS 8.5
GPU	NVIDIA Tesla V100 32GB*2	Pytorch	1.13.1
RAM	64GB	CUDA	11.6
Hard disk	1.8TB	cuDNN	8.9.4
		Python	3.9.19

**Table 4:** Hyperparameter information during training.

Hyperparameters	Value	Hyperparameters	Value
Epoch	400	optimizer	SGD
Batch size	32	momentum	0.937
Num workers	8	learning rate	0.01
Image size	640	weight decay	0.0005

**Table 5:** Precision indicators for ablation experiments.

YOLOv8n	ESPPF	EHConv	RC2f	PFDetect	Precision (%)	Recall (%)	mAP@50 (%)	mAP@50:95 (%)	F1-Score (%)
√					98.9	99	99.4	88.4	98.95
√	√				98.8	98.3	99.2	88.4	98.55
√	√	√			99	98.8	99.4	87.7	98.90
√	√		√		98.9	98.8	99.4	88	98.85
√	√			√	99.1	98.8	99.4	88	98.95
√	√	√	√		98.9	99.2	99.5	87.5	99.05
√	√	√		√	98.9	98.5	99.5	88	98.70
√	√		√	√	97.9	97.9	99.3	88.1	97.90
√	√	√	√	√	98.5	98.8	99.5	87.4	98.65

**Table 6:** Lightweighting parameters for ablation experiments.

YOLOv8n	ESPPF	EHConv	RC2f	PFDetect	Parameters (Million)	FLOPs (G)	Model Size (MB)	Dt (ms)	FPS
√					3.02	8.1	6	10	100.2
√	√				2.86	8.0	5.7	10.7	93.0
√	√	√			2.30	6.9	4.6	11.1	90.6
√	√		√		2.27	7	4.5	11.5	87.4
√	√			√	2.16	5.4	4.3	11.2	89.2
√	√	√	√		1.71	5.9	3.5	11.5	86.8
√	√	√		√	1.60	4.3	3.3	11.5	86.8
√	√		√	√	1.57	4.4	3.2	11.8	84.8
√	√	√	√	√	1.01	3.3	2.1	11.2	89.4

**Table 7:** Comparison of precision indicators.

	Model	Precision (%)	Recall (%)	mAP@50(%)	mAP@50:95(%)	F1-Score (%)
YOLO Series Models	YOLOv5n	99.7	99.6	99.3	87.6	99.65
	YOLOv7-tiny	98.8	99.6	99.6	85.7	99.20
	YOLOv8n	98.9	99	99.4	88.4	98.95
	YOLOv9-t	99	99.1	99.5	88.3	99.05
	YOLO11n	99	98.8	99.5	88	98.90
Modified Backbone	V3-YOLOv8n	98.7	97.9	99.3	87.3	98.30
	V4-YOLOv8n	98.4	97.8	99.2	87.4	98.10
	LCNet-YOLOv8n	98.2	97	99.2	86.7	97.60
	Ghost-YOLOv8n	98.3	97.6	99.4	86.4	97.95
Ours	PFL-YOLO	98.5	98.8	99.5	87.4	98.65

**Table 8:** Comparison of lightweight parameters.

	Model	Parameters (Million)	FLOPs (G)	Model Size (MB)	Dt (ms)	FPS
YOLO Series Models	YOLOv5n	1.84	4.4	3.8	8.7	114.2
	YOLOv7-tiny	6.17	13.5	12	8.8	114
	YOLOv8n	3.02	8.1	6	10	100.2
	YOLOv9-t	3.51	15.3	7.5	30.5	32.8
	YOLO11n	2.59	6.4	5.2	12.2	81.9
Modified Backbone	V3-YOLOv8n	2.19	5.5	4.5	13.2	76
	V4-YOLOv8n	2.15	5.9	4.4	12.7	79.2
	LCNet-YOLOv8n	1.98	5.5	4	10.7	93.5
	Ghost-YOLOv8n	2.03	5.7	4.2	14.9	67.1
Ours	PFL-YOLO	1.01	3.3	2.1	11.2	89.4

**Table 9:** Experimental comparison results for lightweight detection models.

Model	mAP@50(%)	mAP@50:95 (%)	Parameters (Million)	FLOPs (G)	Model Size (MB)
YOLOv5n	99.3	87.6	1.84	4.4	3.8
YOLOv7-tiny	99.6	85.7	6.17	13.5	12
YOLOv8n	99.4	88.4	3.02	8.1	6
YOLOv9-t	99.5	88.3	3.51	15.3	7.5
YOLO11n	99.5	88	2.59	6.4	5.2
PFL-YOLO	99.5	87.4	1.01	3.3	2.1

**Table 10:** A comparison of the heatmap area of concern.

Image	YOLOv8n (Pixel)	V3-YOLOv8n (Pixel)	V4-YOLOv8n (Pixel)	LCNet-YOLOv8n (Pixel)	Ghost-YOLOv8n (Pixel)	PFL-YOLO (Pixel)
(a)	14,225.5	10,408.5	8773.5	7630.5	11,731.5	20,066
(b)	21,853.5	9820	31,813.5	27,929.5	31,675	38,888.5
(c)	29,417	16,438.5	19,069.5	17,426.5	18,812.5	27,417.5
(d)	17,062.5	17,556	6816.5	8082	13,119	13,686

## Data Availability

The data presented in this study are available on request from the corresponding author. As the research has not yet concluded, the dataset has not been made public.
